# Autochthonous Dengue Fever in 2 Patients, Rome, Italy

**DOI:** 10.3201/eid3001.231508

**Published:** 2024-01

**Authors:** Serena Vita, Licia Bordi, Giuseppe Sberna, Priscilla Caputi, Daniele Lapa, Angela Corpolongo, Cosmina Mija, Alessandra D’Abramo, Fabrizio Maggi, Francesco Vairo, Eliana Specchiarello, Enrico Girardi, Eleonora Lalle, Emanuele Nicastri

**Affiliations:** National Institute for Infectious Diseases Lazzaro Spallanzani, Rome, Italy

**Keywords:** dengue fever, autochthonous cases, dengue, dengue virus, DENV, viruses, PCR, antigenomic DENV RNA, zoonoses, Italy

## Abstract

Since August 2023, outbreaks of dengue virus (DENV) infection have occurred in Italy. We report 2 autochthonous case-patients and their extended follow-up. Despite persistent DENV detected in blood by PCR, results for antigenomic DENV RNA were negative after day 5, suggesting that a 5-day isolation period is adequate to avoid secondary cases.

Dengue virus (DENV) infection is the most prevalent arthropodborne viral disease in humans, caused by 4 DENV serotypes widely spread in tropical and subtropical regions and transmitted mainly by *Aedes* mosquitoes (*1*). *Aedes albopictus* mosquitoes colonizing every continent except Antarctica has led to an increase in areas of Europe at risk for *Aedes*-borne viruses (*2*,*3*). During August–October 2023, a total of 68 case-patients who had DENV infection and no travel link were reported in Italy, 36 (53%) in Lombardia and 32 (47%) in Lazio; all had a good clinical condition (*4*,*5*). We report 2 autochthonous case-patients who had DENV infection and prolonged viral shedding during a follow-up period of 28 days after symptoms onset.

On August 31, a 46-year-old man (case-patient 1) and a 48-year-old woman (case-patient 2) who were living in Rome, Italy, and had no history of recent international travel or of yellow fever vaccination were referred to the National Institute for Infectious Diseases L. Spallanzani in Rome for history of fever. Both persons were on holiday during August 14–21. On August 27, eighty km south of Rome, where 1 imported DENV case was previously reported, case-patient 1 had a 2-day history of fever with bilateral conjunctivitis and a face and trunk macular rash, and case-patient 2 had a 1-day history of fever with myalgia and arthralgia. No major concurrent illnesses were present.

At admission, we tested the 2 patients for DENV nonstructural protein 1 (NS1) and IgM and IgG by using fluorimetric rapid assays (Standard F Dengue NS1 Ag FIA and Standard F Dengue IgM/IgG FIA; SD Biosensor, https://www.sdbiosensor.com) (*6*). For both patients, rapid assays were positive for DENV NS1 antigen only, which is considered an early marker for acute DENV infection (*7*). Results for chikungunya virus, HIV, hepatitis B virus, and hepatitis C virus were negative. Hematologic analyses showed platelet values within reference limits but leukopenia (minimum 2,760 cells/mm^3^ for case-patient 1 and 1,850 cells/mm^3^ for case-patient 2; reference range 4,000–11,000 cells/mm^3^) and lymphocytopenia (minimum 750 cells/mm^3^ for case-patient 1 and 230 cells/mm^3^ for case-patient 2; reference range 1,000–4,800 cells/mm^3^).

Case-patient 1 had continuous fever (maximum temperature 38.5°C) until day 8, skin macular rash and lymphopenia until day 9, and lowest platelet level (98,000 cells/mm^3^) on day 9. Case-patient 2 had fever (maximum temperature 38.7°C), headache, myalgia, arthralgia, and lymphopenia until day 7.

We performed molecular and serologic analyses during the 28-day follow-up period (Appendix). DENV-specific reverse transcription PCR on plasma and blood samples collected within 3 days after symptom onset yielded positive results, enabling us to identify a DENV-3 infection (*8*). Plasma samples remained positive until day 9 for case-patient 1 and day 8 for case-patient 2. Blood samples were positive at day 17 for case-patient 1 and day 16 for case-patient 2. Saliva sample results were positive until day 9 for case-patient 1 and day 8 for case-patient 2. Positive urine samples were observed only at day 9 for case-patient 1 and day 16 for case-patient 2. Ocular swab specimens remained negative for both patients. At the end of the 28-day follow-up period, all samples were negative in the DENV molecular assay.

We analyzed serum and saliva samples by using an immunofluorescence assay to detect DENV-3–specific IgM, IgG, and IgA at serologic and mucosal levels (Appendix). IgM appeared in serum samples by day 6 and seroconversion of IgG by day 9 in both case-patients. In saliva, IgM, IgG, and IgA were always negative for case-patient 1, and a positive result was obtained for IgA at day 8 for case-patient 2, suggesting an absent/poor antibody response at the mucosal level for these patients.

To determine whether the DENV genome in plasma/blood samples was associated with active viral replication, we measured levels of antigenomic DENV RNA (negative-strand) (Appendix) by using a DENV type 3–specific forward primer because we considered it to be an indirect marker of ongoing viral replication (*9*). Both patients had antigenomic DENV RNA during the acute phase of infection (i.e., day 3), and case-patient 2 was positive for antigenomic DENV RNA until day 5. Thereafter, despite prolonged viral persistence detected by reverse transcription PCR in plasma/blood until day 16, the antigenomic DENV RNA test results were always negative, suggesting absence of ongoing active viral replication. Patients were discharged at day 9 (case-patient 1) and day 8 (case-patient 2) in good clinical condition.

DENV-infected patients can transmit the virus to *Aedes* mosquitoes if bitten after symptom onset. Therefore, patients should use precautionary measures to reduce the risk for transmission (i.e., sleeping alone) during the first 7 days of febrile illness.

Our results suggest that prolonged viral shedding is not always a marker of ongoing replication in blood, and that the 5-day isolation period might be adequate to prevent transmission (*10*). This observation is relevant for nonendemic countries to limit generation and spread of autochthonous cases.

Appendix Additional information for autochthonous dengue fever in 2 patients, Rome, Italy.
