# Supplementary material for: Autochthonous Dengue Fever in 2 Patients, Rome, Italy
Source: Emerg Infect Dis. 2024 Jan;30(1):183–4. doi: 10.3201/eid3001.231508 (PMC10756386; doi:10.3201/eid3001.231508)
Supplement: Appendix — Additional information for autochthonous dengue fever in 2 patients, Rome, Italy. [file 23-1508-Techapp-s1.pdf]

Article DOI: https://doi.org/10.3201/eid3001.231508

EID cannot ensure accessibility for supplementary materials supplied by authors. Readers who have difficulty accessing supplementary content should contact the authors for assistance.

Autochthonous Dengue Fever in 2 Patients, Rome, Italy

Appendix

Appendix Table 1. Characteristics for dengue case-patient 1 during 28 day follow-up from symptom onset, Rome, Italy*

	Days after symptom onset																	
Characteristic	2			3			6			9			17			28		
Real-time RT-PCR Ct																		
Plasma		22.56			21.03			32.03			38.45			Undetectable			Undetectable	
Plasma negative for RNA		–			24.65			Undetectable			Undetectable			–			–	
Serum		–			24.67			33.9			35.26			Undetectable			Undetectable	
Blood		21.96			–			–			–			31.95			Undetectable	
Blood negative for RNA		–			–			–			–			Undetectable			–	
Urine		–			Undetectable			Undetectable			35.00			Undetectable			Undetectable	
Ocular swab specimen		–			Undetectable			Undetectable			Undetectable			Undetectable			Undetectable	
Saliva		–			30.67			26.93			29.97			Undetectable			Undetectable	
Titers	IgG	IgM	IgA	IgG	IgM	IgA	IgG	IgM	IgA	IgG	IgM	IgA	IgG	IgM	IgA	IgG	IgM	IgA
Serum	–	–	–	<1:2	<1:2	–	1:2	1:320	–	1:160	1:640	–	1:640	1:1,280	–	1:640	1:640	–
Saliva	–	–	–	<1:2	<1:2	<1:2	<1:2	<1:2	<1:2	<1:2	<1:2	<1:2	<1:2	<1:2	<1:2	<1:2	<1:2	<1:2

*Ct, cycle threshold; RT-PCR¸ reverse transcription PCR; –, test not performed..

Appendix Table 2. Characteristics for dengue case-patient 2 during 28 day follow-up from symptom onset, Rome, Italy*

<table>
<tr><th rowspan="2">Characteristic</th><th colspan="18">Result</th></tr>
<tr><th colspan="3">1</th><th colspan="3">2</th><th colspan="3">5</th><th colspan="3">8</th><th colspan="3">16</th><th colspan="3">29</th></tr>
<tr><td>Real-time RT-PCR Ct</td><td colspan="3"></td><td colspan="3"></td><td colspan="3"></td><td colspan="3"></td><td colspan="3"></td><td colspan="3"></td></tr>
<tr><td>Plasma</td><td colspan="3">22.69</td><td colspan="3">22.40</td><td colspan="3">24.55</td><td colspan="3">33.22</td><td colspan="3">Undetectable</td><td colspan="3">Undetectable</td></tr>
<tr><td>Plasma negative for RNA</td><td colspan="3">–</td><td colspan="3">25.65</td><td colspan="3">28.98</td><td colspan="3">Undetectable</td><td colspan="3">–</td><td colspan="3">–</td></tr>
<tr><td>Serum</td><td colspan="3">–</td><td colspan="3">18.61</td><td colspan="3">24.93</td><td colspan="3">35.00</td><td colspan="3">Undetectable</td><td colspan="3">Undetectable</td></tr>
<tr><td>Blood</td><td colspan="3">23.69</td><td colspan="3">–</td><td colspan="3">–</td><td colspan="3">–</td><td colspan="3">31.85</td><td colspan="3">Undetectable</td></tr>
<tr><td>Blood negative for RNA</td><td colspan="3">–</td><td colspan="3">–</td><td colspan="3">–</td><td colspan="3">–</td><td colspan="3">Undetectable</td><td colspan="3">–</td></tr>
<tr><td>Urine</td><td colspan="3">–</td><td colspan="3">Undetectable</td><td colspan="3">Undetectable</td><td colspan="3">Undetectable</td><td colspan="3">28.93</td><td colspan="3">Undetectable</td></tr>
<tr><td>Ocular swab specimen</td><td colspan="3">–</td><td colspan="3">Undetectable</td><td colspan="3">Undetectable</td><td colspan="3">Undetectable</td><td colspan="3">Undetectable</td><td colspan="3">Undetectable</td></tr>
<tr><td>Saliva</td><td colspan="3">–</td><td colspan="3">Undetectable</td><td colspan="3">Undetectable</td><td colspan="3">Undetectable</td><td colspan="3">Undetectable</td><td colspan="3">Undetectable</td></tr>
<tr><td>Titers</td><td>IgG</td><td>IgM</td><td>IgA</td><td>IgG</td><td>IgM</td><td>IgA</td><td>IgG</td><td>IgM</td><td>IgA</td><td>IgG</td><td>IgM</td><td>IgA</td><td>IgG</td><td>IgM</td><td>IgA</td><td>IgG</td><td>IgM</td><td>IgA</td></tr>
<tr><td>Serum</td><td>–</td><td>–</td><td>–</td><td><1:2</td><td><1:2</td><td>–</td><td>1:2</td><td>1:80</td><td>–</td><td>1:80</td><td>1:160</td><td>–</td><td>1:320</td><td>1:320</td><td>–</td><td>1:1,280</td><td>1:160</td><td>–</td></tr>
<tr><td>Saliva</td><td>–</td><td>–</td><td>–</td><td><1:2</td><td><1:2</td><td><1:2</td><td><1:2</td><td><1:2</td><td>1:2</td><td><1:2</td><td><1:2</td><td>1:16</td><td><1:2</td><td><1:2</td><td>1:2</td><td><1:2</td><td><1:2</td><td><1:2</td></tr>
</table>

*Ct, cycle threshold; DSO, days from symptom onset; RT-PCR¸ reverse transcription PCR; –, test not performed..
